# Supplementary material for: Polyketide synthase gene clusters in the ericoid mycorrhizal fungus Oidiodendron maius: genome-wide prediction, expression profiling, and functional characterization of knockout mutants
Source: Front Plant Sci. 2026 Sep 16;17:1924430. doi: 10.3389/fpls.2026.1924430 (PMC13623903; doi:10.3389/fpls.2026.1924430)
Supplement: Supplementary file 1 [file SupplementaryFile1.zip › Supplementary Materials/Supplementary Folder 1.zip!/Oidma1_AssemblyScaffolds_Repeatmasked/knownclusterblast/cluster45/jgi.p_Oidma1_60477_mibig_hits.html]

| MIBiG Protein | Description | MIBiG Cluster | MiBiG Product | % ID | % Coverage | BLAST Score | E-value |
| --- | --- | --- | --- | --- | --- | --- | --- |
| BAC10676.1 | putative\_FAD-monooxygenase | BGC0000821 | Rebeccamycin\_biosynthetic\_gene\_cluster | 38 | 98.9031078611 | 325 | 1.1e-88 |
| CAC93716.1 | putative\_monooxygenase | BGC0000822 | Rebeccamycin\_biosynthetic\_gene\_cluster | 38 | 98.9031078611 | 325 | 1.1e-88 |
| BAC15752.1 | RebC | BGC0000823 | Rebeccamycin\_biosynthetic\_gene\_cluster | 38 | 98.9031078611 | 325 | 1.1e-88 |
| ABC02791.1 | putative\_FAD-monooxygenase | BGC0000809 | AT2433\_biosynthetic\_gene\_cluster | 37 | 98.1718464351 | 309 | 1.1e-83 |
| AEO12712.1 | flavin\_monooxygenase | BGC0001334 | Cladoniamide\_biosynthetic\_gene\_cluster | 37 | 102.193784278 | 307 | 2.3e-83 |
| AKQ20690.1 | RebC-like\_monooxygenase | BGC0001337 | Lazarimide\_biosynthetic\_gene\_cluster | 35 | 98.9031078611 | 304 | 3.4e-82 |
| AKG47162.1 | HysC | BGC0001223 | Hydroxysporine\_biosynthetic\_gene\_cluster | 37 | 99.4515539305 | 299 | 6.4e-81 |
| AGI62219.1 | RebC-like\_monooxygenase | BGC0001335 | Borregomycin\_biosynthetic\_gene\_cluster | 35 | 99.0859232176 | 292 | 1e-78 |
| EWM63062.1 | FAD-binding\_monooxygenase | BGC0000679 | Diazepinomicin\_biosynthetic\_gene\_cluster | 34 | 102.010968921 | 291 | 1.7e-78 |
| ABV98191.1 | monooxygenase\_FAD-binding | BGC0000827 | Staurosporine\_biosynthetic\_gene\_cluster | 36 | 98.1718464351 | 290 | 3e-78 |
| BAF47693.1 | monooxygenase | BGC0000825 | Staurosporine\_biosynthetic\_gene\_cluster | 35 | 98.3546617916 | 289 | 6.6e-78 |
| AEF32100.1 | RebC-like\_monooxygenase | BGC0001333 | BE-54017\_biosynthetic\_gene\_cluster | 36 | 98.1718464351 | 287 | 3.3e-77 |
| EFG04594.1 | Staurosporine\_biosynthesis\_monooxygenase\_StaC | BGC0000826 | Staurosporine\_biosynthetic\_gene\_cluster | 35 | 100.731261426 | 280 | 4e-75 |
| ACN29721.1 | monooxygenase | BGC0000814 | K-252a\_biosynthetic\_gene\_cluster | 36 | 99.268738574 | 279 | 8.9e-75 |
| ABD59214.1 | InkE | BGC0000813 | K-252a\_biosynthetic\_gene\_cluster | 36 | 99.268738574 | 279 | 8.9e-75 |
| ADE22300.1 | putative\_FAD-binding\_monooxygenase | BGC0000279 | Xantholipin\_biosynthetic\_gene\_cluster | 31 | 102.193784278 | 222 | 1.3e-57 |
| CBF80483.1 | FAD-dependent\_monooxygenase\_(Eurofung) | BGC0000959 | Aspyridone\_biosynthetic\_gene\_cluster | 32 | 108.043875686 | 219 | 8.4e-57 |
| AMK51275.1 | Hex18 | BGC0001376 | Hexaricin\_biosynthetic\_gene\_cluster | 30 | 104.204753199 | 216 | 7.1e-56 |
| ABX71124.1 | Lct41 | BGC0000238 | Lactonamycin\_biosynthetic\_gene\_cluster | 30 | 101.096892139 | 205 | 1.3e-52 |
| CCH63734.1 | XiaH\_protein | BGC0000665 | Xiamycin\_biosynthetic\_gene\_cluster | 31 | 110.054844607 | 202 | 1.1e-51 |
| AFK78077.1 | XiaK | BGC0000666 | Xiamycin\_biosynthetic\_gene\_cluster | 31 | 110.054844607 | 200 | 4e-51 |
| AHZ61847.1 | Lom13 | BGC0000240 | Lomaiviticin\_biosynthetic\_gene\_cluster | 30 | 104.021937843 | 198 | 1.5e-50 |
| ABP54636.1 | monooxygenase,\_FAD-binding | BGC0000241 | Lomaiviticin\_biosynthetic\_gene\_cluster | 29 | 106.398537477 | 194 | 3.8e-49 |
| AAA67511.1 | hydroxylase | BGC0000275 | Tetracenomycin\_biosynthetic\_gene\_cluster | 28 | 105.850091408 | 181 | 2.5e-45 |
| AAM33672.1 | putative\_FAD-dependent\_monooxygenase\_GrhO5 | BGC0000230 | Griseorhodin\_biosynthetic\_gene\_cluster | 30 | 86.4716636197 | 175 | 1.4e-43 |
| ADE22302.1 | putative\_FAD-binding\_monooxygenase | BGC0000279 | Xantholipin\_biosynthetic\_gene\_cluster | 28 | 102.742230347 | 175 | 2.4e-43 |
| AGD80608.1 | monooxygenase | BGC0000394 | Naphthyridinomycin\_biosynthetic\_gene\_cluster | 35 | 64.533820841 | 156 | 6.7e-38 |
| ABX71099.1 | Lct16 | BGC0000238 | Lactonamycin\_biosynthetic\_gene\_cluster | 29 | 99.0859232176 | 156 | 8.7e-38 |
| AFU65892.1 | DacO1 | BGC0000216 | Dactylocycline\_biosynthetic\_gene\_cluster | 30 | 99.268738574 | 147 | 5.3e-35 |
| CAE17536.1 | oxygenase | BGC0000210 | Chromomycin\_A3\_biosynthetic\_gene\_cluster | 31 | 99.268738574 | 140 | 5e-33 |
| BAQ25478.1 | monooxygenase | BGC0001288 | Maklamicin\_biosynthetic\_gene\_cluster | 29 | 84.0950639854 | 136 | 7.1e-32 |
| AAZ77700.1 | ChlE3 | BGC0000036 | Chlorothricin\_biosynthetic\_gene\_cluster | 31 | 66.9104204753 | 136 | 9.3e-32 |
| BAQ21949.1 | putative\_FAD-dependent\_oxidoreductase | BGC0001204 | Versipelostatin\_biosynthetic\_gene\_cluster | 33 | 67.8244972578 | 136 | 9.3e-32 |
| ADE34483.1 | SsfO2 | BGC0000269 | SF2575\_biosynthetic\_gene\_cluster | 27 | 84.4606946984 | 127 | 5.7e-29 |
| AAZ78335.1 | OxyL | BGC0000254 | Oxytetracycline\_biosynthetic\_gene\_cluster | 28 | 65.2650822669 | 127 | 5.7e-29 |
| CBH32810.1 | putative\_oxygenase | BGC0000263 | Ravidomycin\_biosynthetic\_gene\_cluster | 31 | 65.9963436929 | 123 | 1.1e-27 |
| CBH32087.1 | putative\_FAD-dependent\_monooxygenase | BGC0000211 | Chrysomycin\_biosynthetic\_gene\_cluster | 29 | 97.2577696527 | 122 | 1.4e-27 |
| ACB37744.1 | FAD-dependent\_oxygenase | BGC0000162 | Tetrocarcin\_A\_biosynthetic\_gene\_cluster | 28 | 95.4296160878 | 122 | 1.8e-27 |
| ACB46484.1 | FAD-dependent\_oxidoreductase | BGC0000082 | Kijanimicin\_biosynthetic\_gene\_cluster | 31 | 65.8135283364 | 121 | 4.1e-27 |
| BAJ52699.1 | putative\_FAD-binding\_monooxygenase | BGC0000222 | FD-594\_biosynthetic\_gene\_cluster | 29 | 64.533820841 | 120 | 6.9e-27 |
| AAL33754.1 | putative\_monooxygenase/hydroxylase | BGC0000421 | Safracin\_biosynthetic\_gene\_cluster | 30 | 64.533820841 | 119 | 1.2e-26 |
| AKD43496.1 | Monooxygenase | BGC0001409 | Dutomycin\_biosynthetic\_gene\_cluster | 28 | 68.7385740402 | 117 | 4.5e-26 |
| AHZ61851.1 | Lom17 | BGC0000240 | Lomaiviticin\_biosynthetic\_gene\_cluster | 30 | 69.1042047532 | 116 | 1e-25 |
| ACN64853.1 | PokO2 | BGC0001061 | Polyketomycin\_biosynthetic\_gene\_cluster | 27 | 102.376599634 | 113 | 6.5e-25 |
| AFU65903.1 | DacO2 | BGC0000216 | Dactylocycline\_biosynthetic\_gene\_cluster | 25 | 82.449725777 | 108 | 3.6e-23 |
| AHE14649.1 | FAD-binding\_monooxygenase | BGC0001336 | Erdasporine\_biosynthetic\_gene\_cluster | 27 | 66.5447897623 | 106 | 1e-22 |
| ABP54640.1 | monooxygenase,\_FAD-binding | BGC0000241 | Lomaiviticin\_biosynthetic\_gene\_cluster | 29 | 64.8994515539 | 96 | 1.4e-19 |
| AAG30198.1 | oxygenase | BGC0000261 | R1128\_biosynthetic\_gene\_cluster | 29 | 66.1791590494 | 94 | 4.1e-19 |
| AHD25942.1 | ChdOII | BGC0000208 | Chelocardin\_biosynthetic\_gene\_cluster | 30 | 36.563071298 | 73 | 7.4e-13 |
| BAJ07846.1 | putative\_oxygenase-reductase | BGC0000232 | Hatomarubigin\_biosynthetic\_gene\_cluster | 34 | 35.1005484461 | 71 | 4.8e-12 |
| ABP54649.1 | monooxygenase,\_FAD-binding | BGC0000241 | Lomaiviticin\_biosynthetic\_gene\_cluster | 29 | 33.0895795247 | 58 | 3.2e-08 |
